# Glycoprotein Pathways Altered in Frontotemporal Dementia With Autoimmune Disease

**DOI:** 10.3389/fimmu.2021.736260

**Published:** 2021-09-01

**Authors:** Fiona Bright, Jared S. Katzeff, John R. Hodges, Olivier Piguet, Jillian J. Kril, Glenda M. Halliday, Woojin Scott Kim

**Affiliations:** ^1^School of Medical Sciences, The University of Sydney, Sydney, NSW, Australia; ^2^Brain and Mind Centre, The University of Sydney, Sydney, NSW, Australia; ^3^School of Psychology, The University of Sydney, Sydney, NSW, Australia

**Keywords:** frontotemporal dementia, autoimmune disease, proteomics, serum, thyroid, glycoprotein, glycome, biomarker

## Abstract

Behavioral variant frontotemporal dementia (bvFTD) is a younger onset form of neurodegeneration initiated in the frontal and/or temporal lobes with a slow clinical onset but rapid progression. bvFTD is highly complex biologically with different pathological signatures and genetic variants that can exhibit a spectrum of overlapping clinical manifestations. Although the role of innate immunity has been extensively investigated in bvFTD, the involvement of adaptive immunity in bvFTD pathogenesis is poorly understood. We analyzed blood serum proteomics to identify proteins that are associated with autoimmune disease in bvFTD. Eleven proteins (increased: ATP5B, CALML5, COLEC11, FCGBP, PLEK, PLXND1; decreased: APOB, ATP8B1, FAM20C, LOXL3, TIMD4) were significantly altered in bvFTD with autoimmune disease compared to those without autoimmune disease. The majority of these proteins were enriched for glycoprotein-associated proteins and pathways, suggesting that the glycome is targeted in bvFTD with autoimmune disease.

## Introduction

Behavioral variant frontotemporal dementia (bvFTD) is a non-Alzheimer’s younger onset neurodegenerative disease with a slow and subtle onset and rapid progression (1, 2). Affected individuals exhibit marked behavioral disturbances (1, 2). bvFTD is biologically complex with different pathological signatures and genetic variants that impact on similar cell types and networks in the brain (1, 2). Therefore, deciphering the precise disease mechanism(s) that give rise to various degenerative proteinopathies in the same neuronal systems in bvFTD remains a significant challenge. As indicated in other neurodegenerative diseases, the immune system and inflammation are involved, with innate immunity extensively investigated in bvFTD. There is evidence of a significantly altered glial landscape in diseased brain regions (3–6), a consistent association of bvFTD with the *HLA* immune loci (7, 8) and disease-causative genes that are associated with inflammation in bvFTD (i.e. *C9ORF72, PGRN, TREM2*), as reviewed in detail elsewhere (9).

Recently, we utilized a discovery proteomics approach to assess serum changes in patients with bvFTD demonstrating significant peripheral changes in calcium ion binding and innate immune pathway proteins (10). In contrast, the involvement of adaptive immunity in bvFTD is poorly understood biologically and remains to be explored. Evidence supporting a role for autoimmunity in bvFTD includes bvFTD-associated genetic variants linked to autoimmune conditions (11, 12), an overrepresentation of autoimmune disease in bvFTD, specifically non-thyroid autoimmune conditions linked to clinical and genetic bvFTD variants (13–15), and the presence of autoantibodies in individuals with bvFTD (16–19).

Autoimmunity is suggested to be an integral part of neurodegeneration (20, 21) with considerable evidence of immune system upregulation in neurodegenerative diseases, implying that the body’s immune system attacks cells of the CNS in a similar way to other autoimmune diseases (22). Indeed, international retrospective studies have demonstrated a significantly higher (80%) risk of dementia in middle-aged individuals with autoimmune diseases (23) and individuals admitted to hospital with an autoimmune disease are 20% more likely to have a subsequent admission for dementia (24).

In this study we investigated alterations in adaptive immunity, firstly by determining the prevalence of autoimmune disease in our patient cohort, and then applying our discovery proteomics approach to determine any blood serum changes in bvFTD patients with and without autoimmune disease.

## Materials and Methods

### Patient Information

The proteomics dataset generated in our previous study (10) was re-analyzed specifically for changes in proteins associated with autoimmune disease. bvFTD patients were from FRONTIER, the frontotemporal dementia clinical research group at the University of Sydney Brain and Mind Centre, and from the ForeFront FTD and motor neuron disease clinic at the University of Sydney Brain and Mind Centre. Each patient in the cohort previously underwent neurological examination including a comprehensive cognitive assessment and structural brain MRI, and met current consensus diagnostic criteria for bvFTD (25). In the present study, the bvFTD cohort specifically were screened for prevalence of a panel of pre-determined autoimmune diseases that were collated from the Australian Society of Clinical Immunology and Allergy (ASCIA) (26) and adapted from previously published studies investigating autoimmune disease in international bvFTD cohorts (13, 14). We compared bvFTD patients with autoimmune disease (N=10) (**Table 1**) to those without autoimmune disease (N=62). Human research ethics approval was granted by the University of New South Wales (approval number: HC12573). All information on the bvFTD cohort and materials and methods relating to proteomics were reported in our previous study (10).

**Table 1:** Demographics of bvFTD patients with autoimmune disease.

	Case	Sex	Age	Autoimmune disease
***Non-thyroid***	1	M	67	DM1
	2	M	68	Celiac
	3	M	59	Psoriasis
	4	M	78	DM1
	5	M	64	Psoriasis
	6	M	78	Psoriasis
	7	M	67	DM1
	8	M	74	Psoriasis + Rheumatoid arthritis
***Thyroid***	9	F	56	Hypothyroidism
	10	M	84	Hyperthyroidism

### Blood Sampling and Proteomics Analysis

The current data is derived from our previous proteomics analysis (10). Briefly, blood samples (9 mL) were collected in tubes (BD Vacutainer SST II Advance Tube #367958), and serum prepared by centrifugation at 3,500 rpm for 10 min at 4°C, which was then aliquoted and stored at −80°C until use. A comprehensive analysis of bvFTD serum proteins using proteomics based on the advanced liquid chromatography-tandem mass spectrometry (LC–MS–MS) technology was then undertaken. Briefly, protein depletion method was used in which 96% of 14 high-abundant proteins (e.g. albumin, IgG) were removed (27) using a 4.6 mm × 100 mm Multiple Affinity Removal System column (MARS, Agilent, Santa Clara, CA, USA) based on the depletion method (27) and following the manufacturer’s instructions, allowing for greater accuracy in identifying less abundant proteins. A total of 40 μl of serum was diluted with 120 μl of buffer A and passed through a 0.22 μm filter and centrifuged at 16,000*g*. The supernatant was injected into a MARS column and the flow through collected. The column was washed with buffer B, to elute the bound proteins, before re-equilibrating with buffer A prior to the next sample. Collected fractions were buffer exchanged into 100 mM TEAB. Following this, we performed nano-capillary liquid chromatography-tandem mass spectrometry (LC-MS-MS) using a Dionex Ultimate 3000 HPLC system (Thermo Fisher Scientific, Waltham, MA, USA) coupled to an in-house fritless nano 75 μm × 30 cm column packed with Repro-Sil Pur 120 C18 stationary phase (1.9 μm, Dr, Maisch GmbH, Germany). Separated compounds were analyzed with an Orbitrap Fusion Tribrid Mass Spectrometer (Thermo Fisher Scientific, Waltham, MA, USA) and a synchronous precursor selection MS3 method (28) was used for data collection. Proteome Discoverer 2.2 (Thermo Fisher Scientific, Waltham, MA, USA) was used to analyze the MS data and the raw mass spectrometry data was processed using MaxQuant (29). Proteomics data are available from the corresponding author upon request.

### Western Blotting

Serum (equal volumes) were heated with sample buffer (3.2% SDS, 32% glycerol, 0.16% bromophenol blue, 100 mM Tris-HCl, pH 6.8, 8% 2-mercaptoethanol), electrophoresed on Criterion Stain-free 4-20% SDS-PAGE gels (Bio-Rad) and transferred onto nitrocellulose membranes at 100 volts for 30 min. The membranes were blocked with TBS containing 5% nonfat dry milk and probed with anti-LOXL3 antibody (mouse monoclonal, 1:1000, Santa Cruz, sc377216) overnight at 4°C. The membranes were then washed three times in TBS containing 0.1% Tween 20 and incubated with horseradish peroxidase-conjugated secondary antibody for 2 h at room temperature. Protein bands were detected using enhanced chemiluminescence and Gel Doc System (Bio-Rad). The blots were stripped and probed for housekeeper proteins transferrin. The signal intensity was quantified using Image Lab (Bio-Rad) and NIH ImageJ software (v1.45s).

### Gene Ontology Analysis

Two gene ontology software programs, Bioprofiling (30) (www.bioprofiling.de, 16 Dec 2019) and STRING (31) v11 (16 Dec 2019), were used to interpret and predict function or pathway on a set of proteins identified by the proteomics analysis. The proteins that were significantly altered were inputted separately into each of the programs following their instructions.

### Statistical Analysis

Statistical analysis on proteomics data was performed as previously described (10). Briefly, protein peak intensities were log2 transformed and any missing values were imputed using the k nearest neighbor algorithm (*impute. knn* function from the *impute* package in R). Following imputation, protein intensities were normalized across batches using the RUV-III (Removing Unwanted Variation-III) algorithm (32). Default parameters from the *RUVIII* function were used. After normalization, any proteins that were originally missing were removed, and samples with replicates averaged. Linear models were fitted using the R/Bioconductor software package limma (33). A design matrix which included age and sex as covariates was used and tested for significance of disease status; neither age nor sex had any effect on protein levels. The Benjamini–Hochberg method was used to control for multiple testing, and proteins with an adjusted *P* < 0.05 were considered to be statistically significant. For western blotting data, statistical analysis was performed using SPSS Statistics software (IBM, Chicago, Illinois), using a univariate analysis (general linear model), with age and sex as covariates, and statistical significance set at *P* < 0.05.

## Results

### Analysis of Serum Proteins Altered in bvFTD With Autoimmune Disease

**Figure 1:**
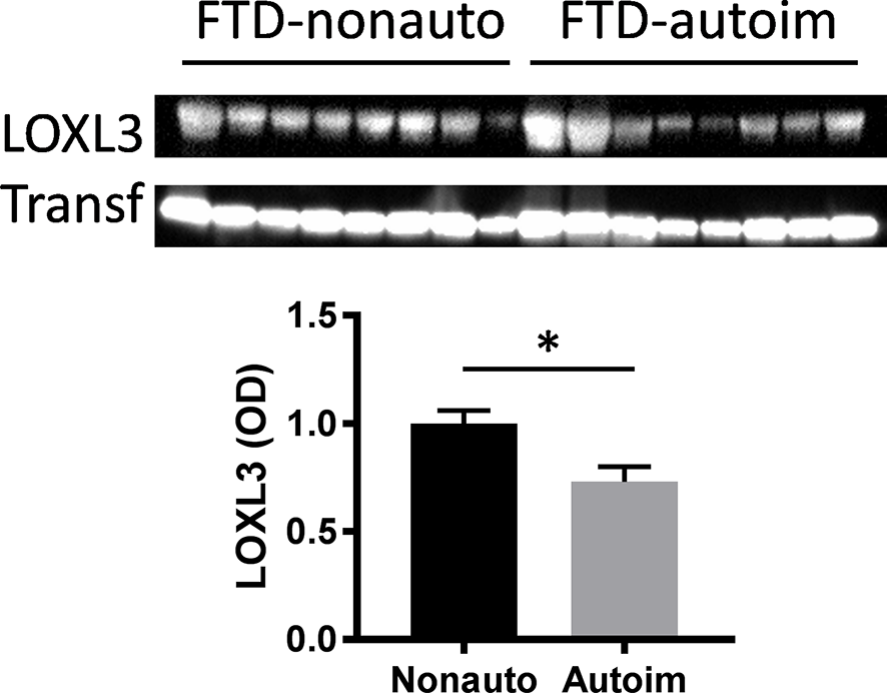
Validation of LOXL3 alteration in bvFTD-autoimmune compared to bvFTD-nonautoimmune serum by western blotting; normalized to the housekeeper protein transferrin (Transf) and optical density (OD) measurements of the bands. Data represent mean and SE as error bars, *P < 0.05.

We analyzed serum proteins in bvFTD patients with autoimmune disease (bvFTD-autoimmune; N=10) (**Table 1**) and bvFTD patients without autoimmune disease (bvFTD-nonautoimmune; N=62) to identify proteins that are associated with autoimmune disease in bvFTD. We found that 11 proteins were significantly altered in bvFTD-autoimmune compared to bvFTD-nonautoimmune covarying for age and sex. Six proteins were significantly increased in bvFTD-autoimmune – ATP5B, CALML5, COLEC11, FCGBP, PLEK, PLXND1; and 5 proteins were significantly decreased – APOB, ATP8B1, FAM20C, LOXL3, TIMD4 (**Table 2**). Although measuring proteins in serum by western blotting is difficult, because of the low sensitivity of interfering proteins, we were able to validate the decrease in LOXL3 in bvFTD-autoimmune by this method (**Figure 1**). Also, we separated non-thyroid autoimmune disease (bvFTD-nonthyroid, N=8) (**Table 1**) from the bvFTD-autoimmune group and compared them to bvFTD-nonautoimmune, and found that 3 proteins were significantly altered; increased – ATP5B, RAB11A; decreased – HRG (**Table 3**).

**Table 2:** Significantly altered proteins in bvFTD-autoimmune compared to bvFTD-nonautoimmune serum.

Protein		Uniprot code	logFC	P value	Highlighted gene ontology molecular and biological functions
*Increased*					
ATP5B*	ATP synthase subunit beta	Q0QEN7	11.9136	0.0133	ATP binding, proton-transporting ATPase activity, ATP synthase, angiostatin binding
PLEK	Pleckstrin	P08567	8.8327	0.0002	Major protein kinase C substrate of platelets, protein kinase C signaling, actin cytoskeleton reorganization
CALML5	Calmodulin Like 5	Q9NZT1	1.4170	0.0349	Calcium ion binding, enzyme regulator activity, signal transduction
COLEC11	Collectin Subfamily Member 11	Q9BWP8	0.8228	0.0133	Innate immunity, apoptosis, embryogenesis, complement activation (lectin pathway)
PLXND1*	Plexin D1	Q9Y4D7	0.8111	0.0349	Protein domain specific binding, semaphoring receptor activity, cell signaling, regulated migration of various cell types
FCGBP	Fc Fragment Of IgG Binding Protein	Q9Y6R7	0.4566	0.0478	Unknown, potential involvement in maintenance of mucosal structure
*Decreased*					
TIMD4*	T Cell Immunoglobulin and Mucin Domain Containing 4	Q96H15	-7.0591	0.0133	Glycoprotein, phosphotidylserine receptor, enhances engulfment of apoptotic cells, regulation of T cell proliferation and lymphotoxin signaling
LOXL3*	Lysyl Oxidase Like 3	P58215	-5.1753	0.0133	Key regulator of glycoproteins, oxioreductase, copper ion binding, fibronectin binding, protein-lysin-6-oxidase activity, scavenger receptor activity, inflammatory response
ATP8B1	ATPase Phospholipid Transporting 8B1	O43520	-1.9138	0.0133	Translocase, ATPase coupled intramembrane lipid transporter activity, lipid transport, ATP binding, binding of magnesium, metal and nucleotides
FAM20C*	FAM20C Golgi Associated Secretory Pathway Kinase	Q8IXL6	-1.1636	0.0133	Transferase, kinase, biomineralization, ATP binding, binding of calcium, manganese and metal, protein serine/threonine kinase activity, phosphotransferase activity
APOB*	Apolipoprotein B	P04114	-0.5062	0.0402	Glycoprotein, heparin binding, cholesterol metabolism, lipid metabolism, lipid transport, steroid metabolism, sterol metabolism, transport, phospholipid binding

*Proteins significantly enriched for UniProt keyword “Glycoprotein”.

**Table 3:** Significantly altered proteins in bvFTD-nonthyroid compared to bvFTD-nonautoimmune serum.

Protein		Uniprot code	logFC	P value	Highlighted gene ontology molecular and biological functions (UniProt)
*Increased*					
RAB11A	RAB11A, Member RAS Oncogene Family	P62491	3.71	0.006	GTPase activity and binding, regulation of intracellular membrane trafficking
ATP5B*	ATP synthase subunit beta	Q0QEN7	9.13	0.007	ATP binding, proton-transporting ATPase activity, ATP synthase, angiostatin binding
*Decreased*					
HRG*	Histidine Rich Glycoprotein	P04196	-0.81	0.020	Plasma glycoprotein, heme binding, angiogenesis, chemotaxis, immunoglobulin binding, metal ion binding

*Proteins significantly enriched for UniProt keyword “Glycoprotein”.

### Predicting Dysregulated Pathways in bvFTD With Autoimmune Disease

**Figure 2:**
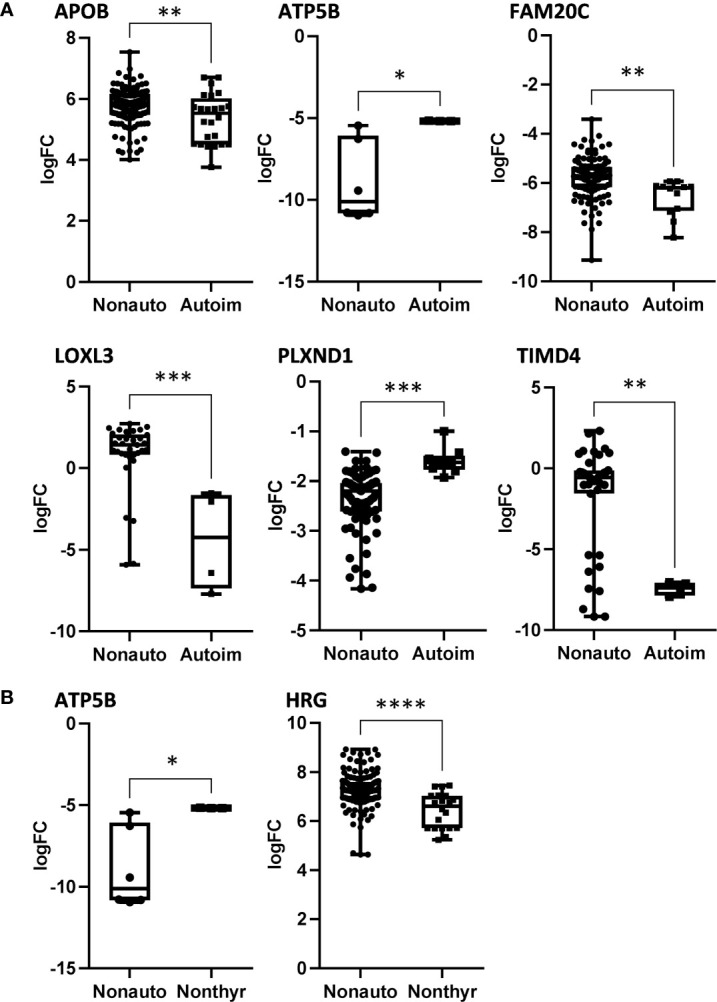
The significantly altered proteins in bvFTD-autoimmune **(A)** and bvFTD-nonthyroid **(B)** compared to bvFTD-nonautoimmune serum that were enriched for the Uniprot keyword “Glycoprotein”. FC, fold change. *P < 0.05, **P < 0.01, ***P < 0.001, ****P < 0.0001.

We then used two gene ontology software programs, STRING and Bioprofiling, to identify or predict functions/pathways possibly altered in bvFTD with autoimmune disease. The 11 proteins altered in bvFTD-autoimmune compared to bvFTD-nonautoimmune were assessed using Bioprofiling. Only one prominent pathway was generated: “Extracellular region” with 5 hits (APOB, FAM20C, FCGBP, LOXL3, PLEK). This same pathway was generated in STRING with the same 5 protein hits (APOB, FAM20C, FCGBP, LOXL3, PLEK). Interestingly, 6 of the 11 altered proteins in bvFTD-autoimmune (APOB, ATP5B, FAM20C, LOXL3, PLXND1, TIMD4) and 2 of the 3 altered proteins in bvFTD-nonthyroid (ATP5B and HRG) (**Figure 2**) were enriched for the Uniprot keyword “Glycoprotein” (KW-0325) and “Immune system process” (GO:0002376).

## Discussion

This study sought to investigate blood serum changes in the context of adaptive immunity in an Australian clinical bvFTD cohort with and without the presence of autoimmune disease. We analyzed our proteomics dataset to determine any blood serum changes in bvFTD with autoimmune disease compared to bvFTD without autoimmune disease. In addition, we determined if there was any change in blood serum in bvFTD with non-thyroid autoimmune disease, given previously published studies reported an increased prevalence specifically of non-thyroid autoimmune conditions in international FTD cohorts (13, 14).

Ten bvFTD individuals (14%) within the cohort were identified to have an autoimmune disease, the majority of which had non-thyroid autoimmune conditions (N=8). In Australia, autoimmune disease occurs in 5% of the population (26) and while our cohort is limited in number, the increased prevalence of autoimmune disease does support previous published findings of an overrepresentation of autoimmune disease in international FTD cohorts (13–15). Interestingly, of these individuals with autoimmune disease, nine were male (90%) and of these bvFTD males with autoimmune disease, all except one had non-thyroid autoimmune conditions. Autoimmune diseases are typically more prevalent in females than males (34, 35) and while analysis of any differences in sex could not be determined given more males than females were identified as having an autoimmune disease in this limited cohort, which is a limitation of the present study, the heightened presence of autoimmune disease in bvFTD males could potentially represent specific adaptive immune alterations driven by the combination of both male sex and autoimmune disease in bvFTD. There is significant evidence demonstrating clear differences between male and female immunity (35, 36) thus potentially a heightened chronic inflammatory environment such as that in neurodegeneration, could further exacerbate sex-related adaptive and peripheral immune responses or vice versa. Further investigation of potential sex-related differences specifically in adaptive immunity within bvFTD is required.

Proteomics analysis of blood serum identified 11 proteins that were significantly altered in bvFTD-autoimmune compared to bvFTD-nonautoimmune participants. While each of the 11 proteins are involved in diverse molecular and biological functions (**Table 2**), gene ontology analysis of these altered proteins identified ‘Extracellular region’ (GO:0005576) as being a significantly enriched pathway in bvFTD-autoimmune serum. Whereas in bvFTD-nonthyroid individuals, only 3 proteins were significantly altered, limiting the ability to perform gene ontology analysis. Of particular interest in the present study is that the majority of the significantly altered proteins were enriched for the Uniprot annotated keyword “Glycoprotein” (KW-0325) and the pathway “Immune system process” (GO:0002376). Glycoproteins play central and diverse roles in inflammatory processes and in the pathophysiology of chronic low-grade inflammatory conditions including diabetes type II, cardiovascular disease and cancer (37, 38). The process of glycosylation, is one of the most essential post-translational modifications (39) and plays a critical role in regulating functional immune responses *via* complex receptor-glycan motif interactions (40, 41). Specifically, protein glycosylation is identified to have a central role in the biochemical stabilization of 3D protein structure, protein folding, protein trafficking on cell membranes as antigen (42). The majority of glycoproteins are localized to the cell surface and are involved in cell adhesion, signal transduction, and structural maintenance of cells and tissues (42).

Glycosylation can be influenced by multiple factors including the type of cell, its activation state, environmental factors, age of the cell and inflammatory mediators such as cytokines (43). Each of these factors can be altered in the setting of disease, therefore glycoprotein expression or in general the ‘glycome’ could represent the overall health status of an individual (38, 44). Indeed, alterations in the human glycome have been associated with cancer and various autoimmune diseases (37, 38). Specifically, glycan processing contributes to the pathogenesis of autoimmune diseases, where abnormal glycosylation of one or more glycoproteins can occur (38). In the adaptive immune system, glycans have important roles in B and T cell differentiation and alterations in glycosylation can modulate inflammatory responses. Importantly, immunoglobulins are themselves glycoproteins, whose biological functions are modulated by their glycosylation patterns. Immunoglobulin glycosylation patterns that are identified to skew the immune system toward a pro- or anti-inflammatory direction are involved in the pathophysiology of autoimmune disease as reviewed in detail elsewhere (38, 45).

In the CNS, glycosylation is vital for maintaining normal brain functions and various glycan-rich molecules within the brain are involved in neural functions, including neuronal development, migration and regeneration (42, 46, 47). In addition, inflammation itself can induce glycan modifications that alter protein folding by masking sites for protease cleavage. This prevents proteolysis and extends the circulating half-life of serum proteins in addition to altering their structure, therefore redirecting the protein to different cell membrane receptors and altering its downstream cellular effects (48, 49). Given inflammation is considered a pathological hallmark of neurodegeneration, it is important to consider what affect this inflammatory response could have on adaptive immunity, particularly given adaptive immunity also serves to support the function of innate immunity and the dialogue between the two is critical and constant. In support of neurodegeneration driving these immune processes, variation in CSF glycan expression has been detected in multiple neurodegenerative diseases, including Alzheimer’s disease (AD), Parkinson’s disease, Huntington’s disease and amyotrophic lateral sclerosis (46, 50–56). Our data adds bvFTD to this list of CNS diseases.

There have been few investigations on the glycome in bvFTD. In AD affected brain regions, there are marked differences in the levels of soluble protein glycans compared to controls, particularly O-GlycNAcytylated and N-O-glycosylated proteins. Of note, individuals with FTD-tau Pick’s disease demonstrate similar lower levels of protein O-GlcNAcylation compared to AD, although they do not have the other AD variations in their glycome. As previously suggested, the glycome of each neurodegenerative disease is likely to differ and should be examined independently (57) as the glycome changes and adapts to maintain optimal function (42, 46, 47).

When comparing both neurodegeneration and autoimmunity, it is important to distinguish that the type of inflammatory/immune response involved in both is different. Autoimmune disease involves adaptive immunity, whereas neurodegenerative disease primarily involves innate immune responses. However, neurodegeneration has been suggested to exist on the same disease spectrum as autoimmunity, despite having different etiologies (20, 21). While the innate immune system has been the focus of investigation in FTD, adaptive immunity has yet to be fully examined, despite evidence to suggest a link between FTD and autoimmunity.

To date, the association between autoimmune disease and FTD has been unified by underlying TDP-43 pathology, and extends to *GRN* carriers (13). Furthermore, GWAS studies have identified novel risk loci that strongly implicate immune pathways in the pathogenesis of TDP-43 specifically (8), and an enrichment of FTD-associated genetic variants is observed in multiple autoimmune disorders (11). While there has been limited investigation into the glycome of sporadic bvFTD specifically, it is important to note that the *GRN* gene is itself a lysosomal glycoprotein critical for proper lysosomal function. Furthermore, *GRN* mutations have also been linked to autoimmunity with multiple studies reporting prominent upregulation of serum GRN levels in individuals with various autoimmune diseases (12, 58–62). In addition, antibodies to GRN have been demonstrated in individuals with histories of particular autoimmune conditions (63). This suggests that bvFTD individuals with *GRN* mutations (not assessed in the present study) in combination with autoimmune disease could have enhanced dysregulation of the glycome. Another bvFTD susceptibility gene triggering receptor expressed on myeloid cells 2 (*TREM2)* located in 6p21.1 MHC/HLA region of the genome is also a cell surface transmembrane glycoprotein (64, 65). Variations in *TREM2* are implicated in autoimmunity and increased risk of autoimmune disease (66, 67).

Given the relationship between these FTD disease causative and susceptibility genes, their involvement in both innate and adaptive immunity, warrants further investigation of the link between glycoproteins and the various FTD subtypes with and without coinciding autoimmune disease. The genetic and pathological attributes of our bvFTD cohort was beyond the scope of the present study, however, in future to determine any specific glycoprotein changes that may be specifically linked to bvFTD and FTD overall further investigation in larger independent cohorts with genetic and pathologically confirmed individuals, with and without autoimmune disease is required.

Despite the need for further investigation and validation, what makes the findings of glycoprotein-associated alterations in bvFTD-autoimmune serum within our cohort particularly interesting, is the clinical utility of assays that are able to measure inflammatory glycoproteins. Unfortunately, sensitive and specific biomarkers of disease for FTD remain elusive which impedes the ability to make accurate diagnosis of the underlying disease subtype during life and also prevents the ability to track disease progression, both of these are critical features required to inform clinical trials. However, in the context of glycoproteins, newly established diagnostic and prognostic tests are able to utilize information from measuring the amount or structure of attached glycans to proteins which could be unique to both the individual and the disease.

In summary, discovery proteomics and gene ontology analysis in serums from bvFTD individuals with (14%) *versus* without autoimmune disease identified numerous glycoprotein-associated serum proteins and pathways that were significantly altered. While an altered glycome is not a new concept in terms of neurodegeneration or autoimmunity, this study provides evidence to suggest that the glycome is particularly affected in individuals with both bvFTD and autoimmune disease. This implies a unique adaptive immune profile specific to bvFTD in the setting of autoimmune disease, either driven by autoimmunity or potentially disease pathogenesis. Future follow up investigation utilizing quantitative glycoproteomics is required in larger independent genetically and pathologically confirmed FTD cohorts to confirm these findings and to further explore potential sex differences that may be associated with bvFTD and autoimmunity. The use of secreted and cell surface glycomes to reflect overall cellular status is routinely assessed (38) and quantified, not only to understand disease mechanisms, but also to improve diagnosis, prognosis and risk prediction (37, 68, 69). Further insights into the structure and function of the glycome in bvFTD could offer an approach for therapeutic development and the ability to fine tune immunological responses and inflammation to optimize the performance of therapeutics specifically targeting bvFTD, as has been suggested for other diseases (38).

## Data Availability Statement

The original contributions presented in the study are included in the article/supplementary material. Further inquiries can be directed to the corresponding author.

## Ethics Statement

The studies involving human participants were reviewed and approved by University of New South Wales Human Research Ethics. The patients/participants provided their written informed consent to participate in this study.

## Author Contributions

WSK designed and supervised the project, analyzed the data, and wrote the manuscript. FB conducted the project, analyzed the data, and wrote the manuscript. JSK conducted the project, analyzed the data, and wrote the manuscript. GMH analyzed the data and revised the manuscript. JJK analyzed the data and revised the manuscript. OP and JRH recruited the patients and performed the neurological examinations, and revised the manuscript. All authors contributed to the article and approved the submitted version.

## Funding

This work was supported by funding to ForeFront, a collaborative research group dedicated to the study of frontotemporal dementia and motor neuron disease, from the National Health and Medical Research Council of Australia (NHMRC) program grant (#1037746). GMH is a NHMRC Senior Leadership Fellow (#1176607) and OP is a NHMRC Senior Research Fellow (#1103258).

## Conflict of Interest

The authors declare that the research was conducted in the absence of any commercial or financial relationships that could be construed as a potential conflict of interest.

## Publisher’s Note

All claims expressed in this article are solely those of the authors and do not necessarily represent those of their affiliated organizations, or those of the publisher, the editors and the reviewers. Any product that may be evaluated in this article, or claim that may be made by its manufacturer, is not guaranteed or endorsed by the publisher.
